# Health Status and Health Care Use Among Adolescents Identified With and Without Autism in Early Childhood — Four U.S. Sites, 2018–2020

**DOI:** 10.15585/mmwr.mm7017a1

**Published:** 2021-04-30

**Authors:** Patrick S. Powell, Karen Pazol, Lisa D. Wiggins, Julie L. Daniels, Gabriel S. Dichter, Chyrise B. Bradley, Rebecca Pretzel, Joy Kloetzer, Charmaine McKenzie, Alexys Scott, Britney Robinson, Amy S. Sims, Eric P. Kasten, M. Daniele Fallin, Susan E. Levy, Patricia M. Dietz, Mary E. Cogswell

**Affiliations:** ^1^National Center on Birth Defects and Developmental Disabilities, CDC; ^2^University of North Carolina at Chapel Hill; ^3^Clinical and Translational Sciences Institute, Michigan State University, East Lansing, Michigan; ^4^Johns Hopkins Bloomberg School of Public Health, Baltimore, Maryland; ^5^Center for Autism Research, Children’s Hospital of Philadelphia.

Persons identified in early childhood as having autism spectrum disorder (autism) often have co-occurring health problems that extend into adolescence (*1*–*3*). Although only limited data exist on their health and use of health care services as they transition to adolescence, emerging data suggest that a minority of these persons receive recommended guidance* from their primary care providers (PCPs) starting at age 12 years to ensure a planned transition from pediatric to adult health care (*4**,**5*). To address this gap in data, researchers analyzed preliminary data from a follow-up survey of parents and guardians of adolescents aged 12–16 years who previously participated in the Study to Explore Early Development (https://www.cdc.gov/ncbddd/autism/seed.html). The adolescents were originally studied at ages 2–5 years and identified at that age as having autism (autism group) or as general population controls (control group). Adjusted prevalence ratios (aPRs) that accounted for differences in demographic characteristics were used to compare outcomes between groups. Adolescents in the autism group were more likely than were those in the control group to have physical difficulties (21.2% versus 1.6%; aPR = 11.6; 95% confidence interval [CI] = 4.2–31.9), and to have additional mental health or other conditions^†^ (one or more condition: 63.0% versus 28.9%; aPR = 1.9; 95% CI = 1.5–2.5). Adolescents in the autism group were more likely to receive mental health services (41.8% versus 22.1%; aPR = 1.8, 95% CI = 1.3–2.6) but were also more likely to have an unmet medical or mental health service need^§^ (11.0% versus 3.2%; aPR = 3.1; 95% CI = 1.1–8.8). In both groups, a small percentage of adolescents (autism, 7.5%; control, 14.1%) received recommended health care transition (transition) guidance. These findings are consistent with previous research (*4**,**5*) indicating that few adolescents receive the recommended transition guidance and suggest that adolescents identified with autism in early childhood are more likely than adolescents in the general population to have unmet health care service needs. Improved provider training on the heath care needs of adolescents with autism and coordination of comprehensive programs^¶^ to meet their needs can improve delivery of services and adherence to recommended guidance for transitioning from pediatric to adult health care.

Data were collected during July 2018–December 2020 from parents and guardians (parents) of adolescents aged 12–16 years (born September 2003–August 2006) who took part in a multisite study during 2007–2011 at ages 2–5 years (*6*). To assess the feasibility of conducting a larger follow-up study of all participants who took part in the multisite study, researchers at four sites (located in Georgia, Maryland, North Carolina, and Pennsylvania) conducted this preliminary follow-up study. Participants had completed key study components and received a final study classification in the autism or control group during the original study, and parents had consented to future follow-up. Participants identified in a second control arm of the original study as having a developmental disability other than autism were excluded from the current analyses due to substantial heterogeneity of conditions. During the original study, classification of adolescents in the autism group was based on a comprehensive in-person evaluation (*7*).

For the current follow-up study, parents completed survey questions and standardized scales related to the adolescent’s daily living skills (*8*), current severity of autism symptoms, overall health status, physical difficulties (e.g., difficulty walking, using hands to write or eat, hearing, or seeing), gastrointestinal symptoms, sleep problems, mental health or other conditions, and use of health and mental health services during the previous 12 months.** Parents were also asked about transition planning in the context of the three elements included in the Health Resources and Services Administration Maternal and Child Health Bureau (MCHB) National Performance Measure. Adolescents were considered to have received the recommended guidance on transition planning if they met all three elements, which include 1) time alone, without a parent present, with PCP at last preventive visit; 2) PCP actively worked with child; and 3) parent knows how child will be insured as he or she becomes an adult.

Categorical sociodemographic variables were compared using a modified Poisson regression with robust error variance to estimate unadjusted prevalence ratios (PRs) and 95% CIs; PRs were considered significant when the 95% CI did not include 1. For all other categorical variables, PRs and 95% CIs were adjusted for demographics (aPR)^††^ to examine differences between the autism and control groups. Continuous variables (e.g., mother’s and adolescent’s age, difficulty carrying out daily living tasks, and autism symptoms) were examined via linear regression. Because between-group differences could be influenced by the higher percentage of co-occurring intellectual disability among adolescents in the autism group, sensitivity analyses were conducted that excluded participants with a current diagnosis of intellectual disability. Additionally, to assess the potential impact of the COVID-19 pandemic, investigators compared use of health care services and transition planning among participants who completed the survey during July 2018–February 2020 to those who completed the survey during March 2020–December 2020. All analyses were conducted using R (version 3.6.1; The R Foundation).

As of December 2020, the survey had been completed by 146 parents of adolescents in the autism group and 249 in the control group.^§§^^,^^¶¶^ Mean age of adolescents was 14.7 years (interquartile range = 14.3–15.0). Compared with the control group, the following percentages were higher among the autism group: mother born outside the United States (17.1% versus 6.0%), household income below the federal poverty level (26.7% versus 11.2%), and use of public insurance only (24.0% versus 6.0%) or both public and private insurance (23.3% versus 3.2%). A higher percentage of adolescents in the autism group were male (80.1% versus 49.0%) and non-Hispanic Black (21.2% versus 9.2%). Adolescents in the autism group demonstrated greater difficulty carrying out daily tasks independently and had higher autism symptom severity scores (Table 1).

**TABLE 1:** Sociodemographic characteristics of mothers and their adolescent children in the autism spectrum disorder and the general population control groups — Study to Explore Early Development, four U.S. sites, 2018–2020*

Adolescent/Maternal characteristic	Autism, % (n = 146)	Control, % (n = 249)	Autism versus control, PR^†^ (95% CI)^§^
**Maternal age, yrs, mean (SD)**	45.8 (6.7)	46.4 (4.6)	p = 0.3^¶^
**Adolescent’s age, yrs, mean (SD)**	14.7 (0.6)	14.7 (0.4)	p = 0.4^¶^
**Maternal education **			
≤High school diploma	7.5	5.6	1.3 (0.6–2.9)
Some college or technical degree	23.3	16.1	1.5 (1.0–2.2)
Bachelor’s degree	33.6	41.4	0.8 (0.6–1.1)
Advanced degree	35.6	36.9	1.0 (0.8–1.2)
Mother born outside United States**	17.1	6.0	2.8 (1.6–5.2)
Adolescent’s sex**			
Female	19.9	51.0	0.4 (0.3–0.6)
Male	80.1	49.0	1.6 (1.4–1.9)
Adolescent’s race/ethnicity**^,††^			
White, non-Hispanic	56.2	76.7	0.7 (0.6–0.9)
Black, non-Hispanic	21.2	9.2	2.3 (1.4–3.8)
Other, non-Hispanic	14.4	7.6	1.9 (1.1–3.4)
Hispanic	8.2	6.4	1.3 (0.6–2.6)
Primary language spoken in home**			
English	93.2	96.8	1.0 (0.9–1.0)
Other	6.8	3.2	2.1 (0.9–5.3)
**Current household income, % FPL^§§^**			
<100	26.7	11.2	2.4 (1.6–3.8)
100–199	16.4	10.8	1.6 (0.9–2.6)
200–299	39.7	58.2	0.7 (0.6–0.9)
≥300	11.6	16.5	0.7 (0.4–1.2)
**Insurance^¶¶^**			
Private only	51.4	90.0	0.6 (0.5–0.7)
Public only	24.0	6.0	4.0 (2.3–7.0)
Both public and private	23.3	3.2	7.3 (3.5–15.2)
Daily living skills,*** mean (SD)	22.7 (7.1)	31.5 (2.6)	p≤0.001
**Autism symptom severity,^†††^ mean (SD)**	70.7 (12.9)	46.8 (8.1)	p≤0.001

**Abbreviations:** CI = confidence interval; FPL = federal poverty level; PR = prevalence ratio; SD = standard deviation.

* Survey data were collected from four sites in Georgia, Maryland, North Carolina, and Pennsylvania as part of a preliminary follow-up study of parents or guardians of adolescents aged 12–16 years who were enrolled in the Study to Explore Early Development (https://www.cdc.gov/ncbddd/autism/seed.html) at ages 2–5 years and initially identified as having autism (autism group) or as general population controls (control group).

^†^ For categorical variables, unadjusted PRs were estimated using a modified Poisson regression with robust standard error (https://doi.org/10.1093/aje/kwh090) and study group (autism or control) as the only predictor variable.

^§^ PRs were considered significant when the 95% CI did not include the null value of 1.

^¶^ For continuous variables (e.g., maternal age, child age, daily living skills, and autism symptom severity), linear regression was conducted using study group (autism or control) as the only predictor variable.

** Data collected as part of original Study to Explore Early Development when child was aged 2–5 years.

^††^ Maternal and paternal race/ethnicity used in combination to assign adolescent race/ethnicity.

^§§^ Data missing for 16 participants (autism: n = 8; control: n = 8).

^¶¶^ Uninsured participants not reported because of small sample size (autism: n = 2; control: n = 2).

*** Current daily living skills measured by Waisman Activities of Daily Living, which contains 17 items; each item is rated as 0 = does not do, 1 = does with help, 2 = does on own. Item scores are summed to produce an overall score; a maximum score of 34 indicates complete independence. https://doi.org/10.1016/j.dhjo.2012.08.005

^†††^ Current autism symptoms measured by Social Responsiveness Scale, 2nd edition parent-report for school-aged children (https://www.carautismroadmap.org/social-responsiveness-scale/). Scores <60 were considered not clinically significant symptoms of autism; scores of 60–65, 66–75, or >76 indicated mild, moderate, or severe deficiencies in reciprocal social behavior associated with autism, respectively.

Compared with the control group, the percentage of adolescents in the autism group whose overall health was reported as excellent was lower (40.4% versus 65.1%), whereas percentages in the autism group were higher for physical difficulties (21.2% versus 1.6%), sleep problems (54.8% versus 40.2%), and additional mental health or other conditions (one or more condition: 63.0% versus 28.9%, and two or more conditions: 41.8% versus 10.8%). The two most common conditions in both groups were more prevalent in the autism group: attention-deficit/hyperactivity disorder (ADHD) (39.7% versus 15.7%) and anxiety (36.3% versus 16.1%). Intellectual disability was more prevalent in the autism group (27.4% versus 0.8%) (Table 2).

**TABLE 2:** Overall health, physical difficulties, and co-occurring mental health or other conditions among adolescent children in the autism spectrum disorder and the general population control groups — Study to Explore Early Development, four U.S. sites, 2018–2020*

Current health-related outcomes	Autism, % (n = 146)	Control, % (n = 249)	Autism versus control, aPR^†^ (95% CI)^§^
**Overall health **	N/A	N/A	p<0.001^¶^
Excellent	40.4	65.1	0.7 (0.5–0.9)
Very good	41.1	24.9	1.7 (1.2–2.4)
Good	15.1	7.6	2.0 (1.1–3.5)
Fair or poor	3.4	2.4	—**
**Physical difficulties, one or more **	21.2	1.6	11.6 (4.2–31.9)
Difficulty using hands	13.7	0.4	20.8 (3.0–143.4)
Difficulty hearing or deafness	5.5	0.4	—**
Difficulty seeing or blindness	5.5	0.4	—**
Difficulty walking or climbing stairs	3.4	1.2	—**
**Gastrointestinal symptoms/difficulties^††^**	19.9	11.6	1.4 (0.7–2.6)
**At least one sleep problem occurring ≥2 times/week** ^§§^	54.8	40.2	1.5 (1.2–1.9)
**Current mental health or other conditions**			
Attention-deficit/Hyperactivity disorder	39.7	15.7	1.7 (1.2–2.6)
Anxiety	36.3	16.1	2.4 (1.6–3.5)
Intellectual disability	27.4	0.8	30.6 (7.4–127.4)
Depression	6.8	6.8	1.0 (0.4–2.2)
Obsessive-compulsive disorder	8.9	2.0	3.5 (1.1–10.8)
Epilepsy or seizure disorder	7.5	0.4	1.8 (0.5–6.2)
Other conditions^¶¶^	4.1	0.4	—**
One or more conditions	63.0	28.9	1.9 (1.5–2.5)
Two or more conditions	41.8	10.8	3.4 (2.2–5.3)

**Abbreviations:** aPR = adjusted prevalence ratio; CI = confidence interval; N/A = not applicable.

* Survey data were collected from four sites in Georgia, Maryland, North Carolina, and Pennsylvania as part of a preliminary follow-up study of parents or guardians of adolescents aged 12–16 years who were enrolled in the Study to Explore Early Development (https://www.cdc.gov/ncbddd/autism/seed.html) at ages 2–5 years and initially identified as having autism (autism group) or as general population controls (control group).

^†^ aPRs were estimated using a modified Poisson regression with robust standard error (https://doi.org/10.1093/aje/kwh090) and study group (autism or control) as the predictor, adjusted for maternal education, maternal country of birth (born inside or outside the United States), adolescent sex (male or female), adolescent race/ethnicity (non-Hispanic White, non-Hispanic Black, non-Hispanic other, or Hispanic), household income as a percentage of federal poverty level, and insurance type (private, public, both, or neither); data on maternal and paternal race/ethnicity that were collected during the original Study to Explore Early Development were used in combination to assign adolescent race/ethnicity.

^§^ aPRs were considered significant when the 95% CI did not include the null value of 1.

^¶^ Significance testing conducted using ordinal logistic regression; p-values indicate significant between group variation.

** aPR suppressed because of small cell size (n<10) and low estimated stability.

^††^ Parents who indicated that during the previous 12 months their child had frequent or chronic difficulty with any digesting food, including stomach or intestinal problems, constipation, or diarrhea.

^§§^ Sleep problems included teeth grinding, restlessness, bed-wetting, sleep talking, sleep walking, nightmares, and night terrors.

^¶¶^ Other conditions included substance abuse, bipolar disorder, Tourette syndrome, fragile X syndrome, or Down syndrome; adolescents with more than one of these specific conditions are represented only once.

The percentage of participants receiving a preventive health check-up within the previous 12 months was 89.7% for the autism group and 96.0% for the control group. Compared with the control group, a higher percentage of participants in the autism group received mental health services (41.8% versus 22.1%) and had an unmet medical or mental health care service need (11.0% versus 3.2%). In both groups, a small percentage of adolescents met all three elements of the health care transition measure (i.e., the child spent time alone with the PCP, without a parent present at last preventive visit; the PCP actively worked with child; and the parent knows how child will be insured as he or she reaches adulthood) (autism, 7.5%; control, 14.1%). The percentage of those receiving a limited number of transition planning elements (i.e., none or one of three) was higher in the autism group (69.2% versus 43.0%), whereas the percentage who spoke with their doctor or PCP privately, without a parent present, was lower (38.4% versus 66.3%) (Table 3).

**TABLE 3:** Health care use, need, and transition planning among adolescent children in the autism spectrum disorder and general population control groups — Study to Explore Early Development, four U.S. sites, 2018–2020*

Health care use and need	Autism, % (n = 146)	Control, % (n = 249)	Autism versus control, aPR^†^ (95% CI)^§^
**Received health care services in previous 12 mos**			
Preventive check-ups^¶^	89.7	96.0	0.9 (0.8–1.0)
Medical care of any type**	93.2	98.0	0.9 (0.9–1.0)
Mental health^††^	41.8	22.1	1.8 (1.3–2.6)
**Needed health care services at any time in previous 12 mos but did not receive**			
Health care of any type^§§^	11.0	3.2	3.1 (1.1–8.8)
Medical care of any type^¶¶^	7.5	2.0	3.4 (1.0–11.8)
Mental health***^,†††^	7.5	3.2	2.2 (0.7–6.6)
**Health care transition components** ^§§§^			
Actively worked with doctor or health care provider^¶¶¶^	23.3	28.1	0.8 (0.5–1.2)
Parents know how child will be insured as an adult	47.7	63.5	0.8 (0.6–1.0)
Child sees doctor or health care provider privately	38.4	66.3	0.6 (0.5–0.8)
**Health care transition components met** ^§§§^			
Met all three components	7.5	14.1	0.7 (0.3–1.5)
Met two or more components	30.8	57.0	0.6 (0.4–0.8)
Met zero or one component	69.2	43.0	1.5 (1.2–1.9)

**Abbreviations**: aPR = adjusted prevalence ratio; CI = confidence interval.

* Survey data were collected from four sites in Georgia, Maryland, North Carolina, and Pennsylvania as part of a preliminary follow-up study of parents or guardians of adolescents aged 12–16 years who were enrolled in the Study to Explore Early Development (https://www.cdc.gov/ncbddd/autism/seed.html) at ages 2–5 years and initially identified as having autism (autism group) or as general population controls (control group).

^†^ aPRs were estimated using a modified Poisson regression with robust standard error (https://doi.org/10.1093/aje/kwh090) and study group (autism or control) as the predictor, adjusted for maternal education, maternal country of birth (born inside or outside the United States), adolescent sex (male or female), adolescent race/ethnicity (non-Hispanic White, non-Hispanic Black, non-Hispanic other, or Hispanic), household income as a percentage of federal poverty level, and insurance type (private, public, both, or neither); data on maternal and paternal race/ethnicity that were collected during the original Study to Explore Early Development were used in combination to assign adolescent race/ethnicity.

^§^ aPRs were considered significant when the 95% CI did not include the null value of 1.

^¶^ One or more preventative check-ups in the previous 12 months.

** Includes any visit to a doctor, nurse or other health care provider for sick-child care, preventive check-ups, physical exams, hospitalizations, or any other medical care.

^††^ Includes adolescents whose parents affirmed that they had received treatment or counseling from a mental health professional in the previous 12 months.

^§§^ Includes adolescents whose parents reported that they needed health care of any type in the previous 12 months but did not receive it. Health care of any type includes medical, dental, vision, hearing, and mental health care.

^¶¶^ Includes adolescents whose parents affirmed the types of care that they specifically needed (i.e., medical, dental, vision, or hearing care) in the previous 12 months but did not receive it.

*** Includes adolescents whose parents indicated that they needed treatment or counseling from a mental health professional but did not receive it.

^†††^ Data missing from one participant in the autism group.

^§§§^ Adolescents met the National Performance Measure of the Health Resources and Services Administration Maternal and Child Health Bureau (https://mchb.tvisdata.hrsa.gov/PrioritiesAndMeasures/NationalPerformanceMeasures) if all three elements of the health care transition measure (https://doi.org/10.1007/s10995-019-02858-6) were met.

^¶¶¶^ This element comprised four indicators. Parents were asked whether their child’s doctors or primary care providers actively worked with the child to 1) think about and plan for his/her future; 2) make positive choices about his/her health; 3) gain skills to manage his/her health and health care; and 4) understand the changes in health care that happen at age 18 years. To meet criteria for this component, the adolescent’s parent had to endorse at least three of four indicators.

Results were similar when adolescents with a current intellectual disability diagnosis were excluded. Adolescents in the autism group had more physical difficulties and additional mental health or other conditions than did those in the control group (Supplementary Table 1, https://stacks.cdc.gov/view/cdc/105176) and completed fewer transition planning components (Supplementary Table 2, https://stacks.cdc.gov/view/cdc/105177). Comparing participants who completed the survey before and after the onset of the COVID-19 pandemic, investigators noted no significant declines in the receipt of services or health care transition planning elements.

## Discussion

This study confirms previous research indicating that physical difficulties and co-occurring mental health or other conditions are prevalent among adolescents identified with autism in early childhood (*1*–*3*,*9*). Approximately one in five had physical difficulties, and approximately three in five had additional mental health or other conditions, such as ADHD or anxiety. Compared with adolescents in the control group, those with autism were 90% more likely to have additional mental health or other conditions, yet three times more likely to have an unmet health care service need. Consistent with other studies (*4*,*5*,*10*), a small percentage of adolescents in both groups (7.5% versus 14.1%) received the recommended guidance on health care transition planning from their doctors or health care providers. However, the percentage of adolescents receiving little to no transition guidance from their PCPs (i.e., none or one of three transition recommendations) was higher in the autism group. Taken together with other studies (*1*–*3*,*9*,*10*), these results not only add to the growing body of evidence indicating a gap in transition guidance for adolescents in the general population but also suggest that adolescents with autism are even less likely to receive this guidance.

The findings in this report are subject to at least five limitations. First, autism was reported but not re-confirmed in adolescents, so some adolescents who were identified with autism in early childhood might no longer meet the criteria applied in the original study (*7*). Second, not all eligible participants responded, which might have led to selection bias. Third, the small sample size limited statistical power. Fourth, data are based on parent-report and might be subject to recall or social desirability bias. Finally, although relevant demographic characteristics were adjusted for when computing aPRs, residual confounding possibly remained.

The findings provided in this report indicate that adolescents with autism had greater physical difficulties, had poorer physical and mental health, and experienced greater gaps in health care use and transition planning than did adolescents from the population control group. Potential strategies for improving health outcomes and reducing gaps in use of services for adolescents with autism include offering interdisciplinary training to professionals that promotes use of evidence-based interventions and increases provider comfort in treating adolescents with autism and other developmental disorders;*** improving delivery of care to be timely, coordinated, and family-centered;^†††^ and promoting programs that facilitate successful health care transition for adolescents, including those with autism and other developmental disorders.^§§§^

**Summary**

**What is already known about this topic?**

Mental health and other conditions are more frequent among children with autism; these conditions often persist into adolescence and require more services and coordination of care.

**What is added by this report?**

Compared with a general population control group, adolescents with autism were 90% more likely to have additional mental health or other conditions and three times more likely to have unmet health care service needs.

**What are the implications for public health practice?**

Improved provider training on the heath care needs of adolescents with autism and coordination of comprehensive programs to meet their needs can improve delivery of services and adherence to guidance for transitioning from pediatric to adult health care.

## Footnotes

* Recommended guidance on health care transition is defined in the context of three transition elements included in the National Performance Measure of the Health Resources and Services Administration (HRSA) Maternal and Child Health Bureau (MCHB) (https://mchb.tvisdata.hrsa.gov/PrioritiesAndMeasures/NationalPerformanceMeasures). The three elements are 1) time alone, without a parent present, with PCP at last preventive visit; 2) PCP actively worked with child; and 3) parent knows how child will be insured as he or she becomes an adult. Adolescents met the health care transition measure if all three elements were endorsed by the adolescent's parent. https://doi.org/10.1007/s10995-019-02858-6

† Mental health and other conditions included attention-deficit/hyperactivity disorder, anxiety, intellectual disability, depression, obsessive-compulsive disorder, epilepsy/seizure disorder, bipolar disorder, substance abuse disorders, Tourette syndrome, fragile X syndrome, and Down syndrome.

§ Affirmative response to the question, “During the past 12 months, was there any time when this child needed health care, but it was not received? By health care, we mean medical care as well as other kinds of care like dental care, vision care, and mental health services.”

¶ For example, HRSA MCHB’s adolescent and young adult programs. https://mchb.hrsa.gov/maternal-child-health-topics/adolescent-and-young-adult-health

** Measures used to assess health-related outcomes and use of services included 1) daily living skills: Waisman Activities of Daily Living (https://doi.org/10.1016/j.dhjo.2012.08.005); 2) autism symptoms: Social Responsiveness Scale 2nd edition parent-report for school-aged children form (https://www.carautismroadmap.org/social-responsiveness-scale/) wherein total standard scores <60 were considered not clinically significant symptoms of autism spectrum disorder; 3) overall health: parents rated child’s health as excellent, very good, good, fair, or poor; 4) physical difficulties: parents indicated if child had any difficulty with walking or climbing stairs, using his or her hands (e.g., for writing or eating), hearing or deafness, or seeing or blindness; 5) gastrointestinal symptoms: parents indicated if child had any frequent or chronic difficulty in the previous 12 months with digesting food, including stomach or intestinal problems, constipation, or diarrhea; 6) sleep problems: parents indicated that their child experienced one or more of the following sleep problems at least two or more times per week: teeth grinding, restlessness, bed-wetting, sleep talking, sleep walking, nightmares, and night terrors; and 7) health and mental health services, which included preventive check-ups (including well-child visits), medical care of any type (including seeing a doctor, nurse, or other health care professional for sick-child care, physical exams, or hospitalizations), and mental health care (including seeing a psychiatrist, psychologist, psychiatric nurse, or clinical social worker).

†† PRs adjusted for mother’s level of education, mother’s country of birth, adolescent’s sex, adolescent’s race and ethnicity, income relative to the federal poverty level, and insurance type. Additional details on demographics are provided (Table 1).

§§ A total of 1,007 adolescents met the eligibility criteria for the follow-up study. As of December 2020, approximately 82% (824) had been invited to participate, 70% (581) of those invited were enrolled, and 68% (395) of those enrolled completed the follow-up study. Participation rates for the autism versus control group did not differ for either the percentage of those invited who enrolled (67% versus 73%; p = 0.3) or the percentage completing the study (61% versus 73%; p = 0.1).

¶¶ Demographic data from the original study indicated that parents who participated in the follow-up study, compared with those who did not, were more likely to have a bachelor’s degree or higher degree (71.8% versus 56.0%; PR = 1.6; 95% CI = 1.3–1.9) and have an annual income of $70,000 or more (63.6% versus 53.7%; PR = 1.3; 95% CI = 1.1–1.5). Adolescents in the current study were more likely to be non-Hispanic White (69.1% versus 56.3%; PR = 1.2; 95% CI = 1.1–1.4). Mother's primary language and country of birth did not differ between those who did and did not participate in the current study.

*** For example, HRSA MCHB’s Autism Initiatives. https://mchb.hrsa.gov/maternal-child-health-initiatives/autism

††† For example, the Association of University Centers on Disabilities Children's Mental Health Champions program: https://www.aucd.org/template/news.cfm?news_id=14854&parent=16

§§§ For example, HRSA MCHB’s Center for Health Care Transition. https://www.hrsa.gov/library/got-transition
